# Resveratrol Attenuates the Na^+^-Dependent Intracellular Ca^2+^ Overload by Inhibiting H_2_O_2_-Induced Increase in Late Sodium Current in Ventricular Myocytes

**DOI:** 10.1371/journal.pone.0051358

**Published:** 2012-12-13

**Authors:** Chunping Qian, Jihua Ma, Peihua Zhang, Antao Luo, Chao Wang, Zhiqiang Ren, Linghao Kong, Shuo Zhang, Xiaojing Wang, Ying Wu

**Affiliations:** Cardio-Electrophysiological Research Laboratory, Medical College, Wuhan University of Science and Technology, Wuhan, Hubei, People’s Republic of China; University of Toronto, Canada

## Abstract

**Background/Aims:**

Resveratrol has been demonstrated to be protective in the cardiovascular system. The aim of this study was to assess the effects of resveratrol on hydrogen peroxide (H_2_O_2_)-induced increase in late sodium current (*I*_Na.L_) which augmented the reverse Na^+^-Ca^2+^ exchanger current (*I*_NCX_), and the diastolic intracellular Ca^2+^ concentration in ventricular myocytes.

**Methods:**

*I*_Na.L_, *I*_NCX,_ L-type Ca^2+^ current (*I*_Ca.L_) and intracellular Ca^2+^ properties were determined using whole-cell patch-clamp techniques and dual-excitation fluorescence photomultiplier system (IonOptix), respectively, in rabbit ventricular myocytes.

**Results:**

Resveratrol (10, 20, 40 and 80 µM) decreased *I*_Na.L_ in myocytes both in the absence and presence of H_2_O_2_ (300 µM) in a concentration dependent manner. Ranolazine (3–9 µM) and tetrodotoxin (TTX, 4 µM), *I*_Na.L_ inhibitors, decreased *I*_Na.L_ in cardiomyocytes in the presence of 300 µM H_2_O_2_. H_2_O_2_ (300 µM) increased the reverse *I*_NCX_ and this increase was significantly attenuated by either 20 µM resveratrol or 4 µM ranolazine or 4 µM TTX. In addition, 10 µM resveratrol and 2 µM TTX significantly depressed the increase by 150 µM H_2_O_2_ of the diastolic intracellular Ca^2+^ fura-2 fluorescence intensity (FFI), fura-fluorescence intensity change (△FFI), maximal velocity of intracellular Ca^2+^ transient rise and decay. As expected, 2 µM TTX had no effect on *I*_Ca.L_.

**Conclusion:**

Resveratrol protects the cardiomyocytes by inhibiting the H_2_O_2_-induced augmentation of *I*_Na.L._and may contribute to the reduction of ischemia-induced lethal arrhythmias.

## Introduction

Despite intensive research has been conducted in recent years, cardiac arrhythmias remain a serious problem. Late sodium current (*I*_Na.L_) has been recognized as an important factor contributing to the abnormal repolarization in ischemic and failured hearts [1]. *I*_Na.L_ plays an important role in determining the action potential duration (APD) [2] and the alteration of the intracellular Na^+^ concentration ([Na^+^]_i_) [3], [4]. It has also been reported that hypoxia increased *I*_Na.L_ in rat ventricular myocytes [4], and the increase in Na^+^ inflow during hypoxia increased [Na^+^]_i_ which in turn rose the intracellular Ca^2+^ concentration ([Ca^2+^]_i_) via the Na^+^-Ca^2+^ exchanger (NCX) resulting in a Na^+^-dependent intracellular Ca^2+^ overload induced by *I*_Na.L_ [5], [6], [7]. An increase in [Ca^2+^]_i_ caused cardiac arrhythmias and irreversible cell damage [8]. Furthermore, increased *I*_Na.L_ caused arrhythmic activity and contractile dysfunction [9], [10]. Therefore, inhibition of *I*_Na.L_ is considered to be a new potential target for therapeutic intervention in patients with myocardial ischaemia and heart failure [10]–[14].

Resveratrol (trans-3, 4′, 5-trihydroxystilbene), a polyphenol in various vegetables and fruits, is abundant in grapes. The root extracts of Polygonum cuspidatum, a constituent of Chinese and Japanese folk medicine, is also a good source of resveratrol [15]. Sufficient clinical and epidemiological evidence showed that the consumption of red wine reduced the incidence of mortality and morbidity in patients with coronary heart disease [16]. Among all the evidence, the well-known one is now popularly termed as the “French paradox” [16], [17]. Resveratrol has been considered to be responsible for the cardiovascular benefits after moderate wine consumption [18]. It is speculated that resveratrol may act as an antioxidant, which modulates the vascular cell functions [19], inhibits platelet aggregation [20], and reduces lipoprotein oxidation [21], to serve as a cardioprotective agent. H_2_O_2_, a reactive oxygen species, is a by-product of oxidative metabolism in which energy activation and electron reduction are involved, and was enhanced during ischemia-reperfusion of the heart [22]. Excessive amount of H_2_O_2_ augmented *I*_Na.L_ in ventricular myocytes [10], [23], but the reducing agents, e.g., dithiothreitol (DTT) and glutathione (GSH), reversed these changes induced by either H_2_O_2_ or hypoxia [24], [25]. Since resveratrol acts as an antioxidant [26], we presumed that it might inhibit the increase in *I*_Na.L_ induced by H_2_O_2_.

To further clarify the pharmacological mechanisms and the scope of application of the agent, it is critical to determine the effect of resveratrol on *I*_Na.L_. Previous investigation showed that 50 µM of resveratrol reduced *I*_Na.L_ in a recombinant expression system with the R1623Q LQT3 mutation [27]. To our knowledge, the effect of resveratrol on *I*_Na.L_ in ventricular myocytes with increased H_2_O_2_ has not been reported. Therefore, this study was designed to address the impact of resveratrol on the Na^+^-dependent Ca^2+^ overload induced by H_2_O_2_-induced increase in *I*_Na.L_ in ventricular myocytes, with the intention to shed some light on its potential clinical application in the future.

## Materials and Methods

### Isolation of Ventricular Myocytes

Adult New Zealand white rabbits (body weight 1.7–2 kg) of either sex were heparinized (2000 U) and anesthetized with ketamine (30 mg kg^ −1^ i.v.) and xylazine (7.5 mg kg^−1^ i.m.). Hearts were excised rapidly and perfused retrogradely on a Langendorff apparatus for 5 min with a Ca^2+^-free Tyrode’s solution containing (in mM): NaCl 135, KCl 5.4, MgCl_2_ 1, NaH_2_PO_4_ 0.33, HEPES 10 and glucose 10 (pH 7.4, adjusted with NaOH), and then a Tyrode’s solution containing enzyme (collagenase type I, 0.1 g/l) and bovine serum albumin (BSA, 0.5 g/l) for 40–50 min. The perfusate was finally switched to KB solution containing (in mM): KOH 70, taurine 20, glutamic acid 50, KCl 40, KH_2_PO_4_ 20, MgCl_2_ 3, EGTA 0.5, HEPES 10, and glucose 10 (pH 7.4), for 5 min. All perfusates were bubbled with 100% O_2_ and maintained at 37°C. The left ventricles were then cut into small chunks and gently agitated in KB solution. The cells were filtered through nylon mesh and stored in KB solution at 25°C. The use of animals in this investigation was approved by the Institutional Animal Care and Use Committee of Wuhan University of Science and Technology and conformed to the "Guide for the Care and Use of Laboratory Animals" published by the National Institutes of Health (NIH publication no. 85-23, revised 1996) and the Guide for the Care and Use of Laboratory Animals of Hubei Province, China.

### Protocol of Experiments

Isolated cells were perfused with Tyrode's solution saturated oxygenated with 100% O_2_ (control) and were then exposed to Tyrode's solution containing 300 µM H_2_O_2_ for 7 min. Next, isolated cells were perfused with Tyrode's solution containing both 300 µM H_2_O_2_ and one of the following, resveratrol (10, 20, 40 or 80 µM) for 10 min, 4 µM tetrodotoxin (TTX) for 10 min, or 4 µM ranolazine for 5 min.

### Solutions and Drugs

To record *I*_Na.L_, the intracellular pipette solution contained (mM): CsCl 120, CaCl_2_ 1, MgCl_2_ 5, Na_2_ATP 5, TEACl 10, EGTA 11, HEPES 10 (pH 7.3). The extracellular solution contained (mM): NaCl 135, CsCl 70, CaCl_2_ 1,MgCl_2_ 1, CdCl_2_ 0.05, glucose 5, HEPES 5 (pH 7.4). In addition, 1 µM nicardipine was used to block the L-type Ca^2+^ channels.

To record Na^+^-Ca^2+^ exchanger current (*I*_NCX_), the intracellular pipette solution contained (mM): NaCl 20, CaCl_2_ 10, aspartic acid 50, MgCl_2_ 3, EGTA 20, HEPES 10, MgATP 5 and CsOH 120 (pH 7.3). The bath solution contained (mM): NaCl 140,CaCl_2_ 2, MgCl_2_ 2, HEPES 5, and glucose 10 (pH 7.4). In addition, 20 µmol L^−1^ ouabain, 1 mmol L^−1^ BaCl_2_, 2 mmol L^−1^ CsCl and 1 µmol L^−1^ nicardipine were used to block the Na^+^-K^+^ pump, K^+^ channels and L-Ca^2+^ channels, respectively. *I*_NCX_ was measured as the Ni^2+^ sensitive current that could be blocked by NiCl_2_ 5.0 mmol L^−1^.

Krebs-Henseleit bicarbonate (KHB) buffer for intracellular Ca^2+^ fluorescence measurement, the bath solution contained (mM): NaCl 131, KCl 4, CaCl_2_ 1, MgCl_2_ 1, glucose 10, and HEPES 10 (pH 7.4).

To record L-type calcium current (*I*_Ca.L_), the intracellular pipette solution contained (mM): CsCl 80, CsOH 60, aspartic acid 40, CaCl_2_ 0.65, HEPES 5, EGTA 10, MgATP 5 and disodium creatine phosphate 5 (pH 7.2). The bath solution contained (mM): NaCl 135, KCl 5.4, MgCl_2_ 0.5, CaCl_2_ 1.8, NaH_2_PO_4_ 0.33, HEPES 10, glucose 10 (pH 7.4).

H_2_O_2_ was a product of Wuhan Zhongnan Chemical Reagent Co. (Wuhan, China). All other chemicals were purchased from Sigma. Stock solutions of drugs were prepared in water. Each of the stocks was diluted to the required concentrations in the external recording solution immediately before use.

### Electrical Recordings

Experiments were performed at room temperature (22–24°C). Rabbit ventricular myocytes were placed into a recording chamber that was bathed with normal extracellular solution, in the absence and presence of drug (s), at a rate of 2 ml min^−1^. *I*_Na.L_, *I*_NCX_ and *I*_Ca.L_ were recorded in voltage *clamp* mode by using whole-cell patch-clamp techniques in rabbit ventricular myocytes. Patch electrodes were pulled with a two-stage puller (PP-830, Narishige Group, Tokyo, Japan). Their resistances were in the range of 1–1.5 MΩ. Capacitance and series resistances were adjusted to obtain minimal contribution of the capacitive transients. A 60% to 80% compensation of the series resistance was usually achieved without ringing. Currents were obtained with an EPC 9 amplifier (Heka Electronic, Lambrecht, Pfalz, Germany) and a Multiclamp 700B amplifier (Axon Instruments, Inc. USA), filtered at 2 kHz, digitized at 10 kHz, and stored on a computer hard disk for further analysis.

### Intracellular Ca^2+^ Fluorescence Measurement

Myocytes were loaded with fura-2-AM (0.5 µM) for 10 min at 25°C, and fluorescence measurements were recorded with a dual excitation fluorescence photomultiplier system (Ionoptix). Myocytes were imaged through an Olympus IX-70 Fluor 40 × oil objective. The cells were field stimulated with a suprathreshold (150%) voltage and at a frequency of 0.5 Hz, 3-ms duration, using a pair of platinum wires placed on opposite sides of the chamber connected to a FHC stimulator (Brunswick, NE, USA). The polarity of the stimulatory electrodes was reversed frequently to avoid possible build up of electrolyte by-products. Cells were exposed to light emitted by a 75-W lamp and passed through either a 340- or 380-nm filter (bandwidths were ±15 nm) while being stimulated to contract at 0.5 Hz. Fluorescence emissions were detected between 480 and 520 nm by a photomultiplier tube after first illuminating the cells at 340 nm for 0.5 s then at 380 nm for the duration of the recording protocol (333 Hz sampling rate). The 360 excitation scan was repeated at the end of the protocol, and qualitative changes in intracellular Ca^2+^ level were inferred from the ratio of the fura-fluorescence intensity (FFI) at both wavelengths. Intracellular Ca^2+^ fluorescence measurements were assessed using the following indices: diastolic intracellular Ca^2+^ level (diastolic FFI) (340/380 ratio), electrically stimulated rise in intracellular Ca^2+^ (△FFI) (340/380 ratio), maximal velocity of Ca^2+^ rise and Ca^2+^ decay (340/380 ratio).

### Data Analysis

Whole-cell recordings were analyzed using clampfit 9.0 (Axon Instruments, Inc.USA) and PulseFit (V8.74, HEKA). Figures were plotted by Origin (V7.0, OriginLab Co., MA, USA). All amplitudes of *I*_Na.L_ were tested at 200 ms in depolarization testing pulse to eliminate the influence of transient sodium current (*I*_Na.T_). Statistical significance between two groups and multiple groups were evaluated by Student’s t-test and one-way analysis of variance (ANOVA), respectively. All values were expressed as mean ± SD, and the number of cells (n) in each group was given. P<0.05 was considered to be statistically significant.

## Results

### Effects of Resveratrol and TTX on *I*_Na.L_ Under Normal Condition

**Figure 1:**
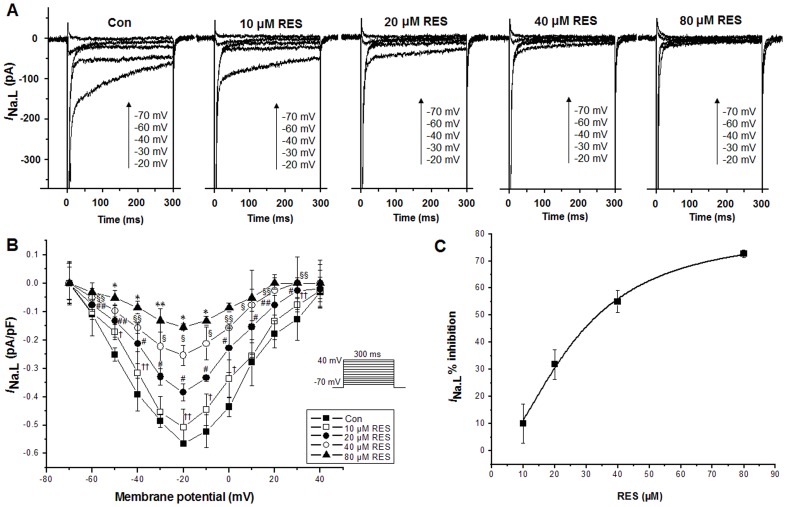
Effects of resveratrol (RES) on *I*_Na.L_ under control condition (Con) in rabbit ventricular myocytes. *A.* 10, 20, 40 and 80 µM resveratrol decreased the amplitude of *I*_Na.L_ in a concentration dependent manner. *B*. Effect of resveratrol (10, 20, 40 and 80 µM) on the current-voltage relationship. *C.* The inhibition amounts of 10, 20, 40 and 80 µM resveratrol on *I*_Na.L_. Values are expressed as mean ± SD, n = 8 cells/group. ^†^P<0.01, ^††^P<0.05 versus control group; ^#^P<0.01, ^##^P<0.05 versus 10 µM resveratrol group; ^§^P<0.01, ^§§^P<0.05 versus 20 µM resveratrol group; *P<0.01, **P<0.05 versus 40 µM resveratrol group.

To identify *I*_Na.L_, the current was recorded first in the absence and then in the presence of 4 µM TTX with 300 ms voltage steps from a holding potential (HP) of −120 to −20 mV. The values of current recorded before (control condition) and after application of TTX were −0.400±0.050 and −0.154±0.038 pA pF^−1^ (n = 6, P<0.05 versus control), respectively, indicating that this TTX-sensitive current recorded was *I*_Na.L_. When *I*_Na.L_ was recorded under normal condition using depolarizing pulses with a duration of 300 ms applied at 0.25 Hz from a HP of −120 mV in 10 mV increments between −70 and −20 mV, administration of 10, 20, 40 and 80 µM resveratrol resulted in decreased amplitudes of *I*_Na.L_ in a concentration dependent manner (Figure 1A, 1B). Figure 1B showed the I-V relationship of *I*_Na.L_ after the administration of 10, 20, 40 and 80 µM resveratrol, without a shift of the voltage at which the *I*_Na.L_ amplitude was maximal (Figure 1B). Figure 1C shows the inhibition amounts of 10, 20, 40 and 80 µM resveratrol on the *I*_Na.L_ with an IC_50_ of 34.442 µM.

### Effects of Resveratrol, Ranolazine and TTX on the Increased *I*_Na.L_ by H_2_O_2_

**Figure 2:**
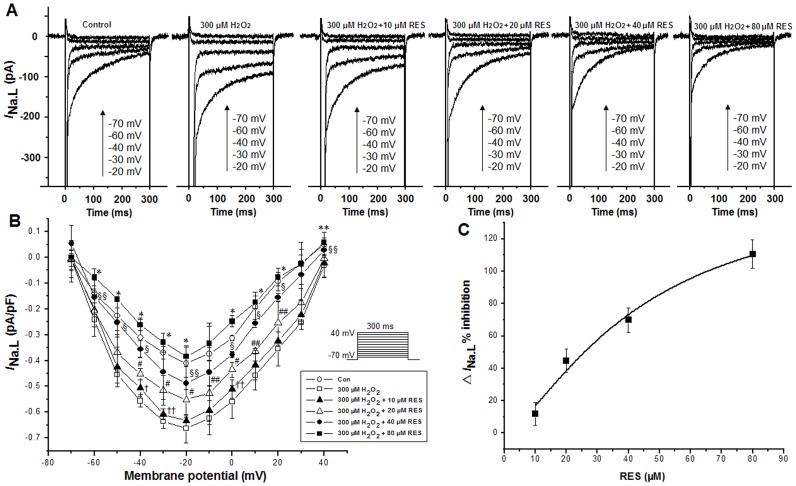
Resveratrol inhibited the increase in *I*_Na.L_ induced by 300 µM H_2_O_2_. *A*. 10, 20, 40 and 80 µM resveratrol decreased the amplitudes of *I*_Na.L_ in the presence of 300 µM H_2_O_2_ in a concentration dependent manner. *B*. The I-V relationships of *I*_Na.L_ after the sequential application of 300 µM H_2_O_2_, 10, 20, 40 and 80 µM resveratrol. *C.* the inhibition amounts of 10, 20, 40 and 80 µM resveratrol on the △*I*_Na.L_ induced by 300 µM H_2_O_2_. Values are expressed as mean ± SD, n = 8 cells/group. ^†^P<0.01, ^††^P<0.05 versus H_2_O_2_ group; ^#^P<0.01, ^##^P<0.05 versus 10 µM resveratrol group; ^§^P<0.01, ^§§^P<0.05 versus 20 µM resveratrol group; *P<0.01, **P<0.05 versus 40 µM resveratrol group.

**Figure 3:**
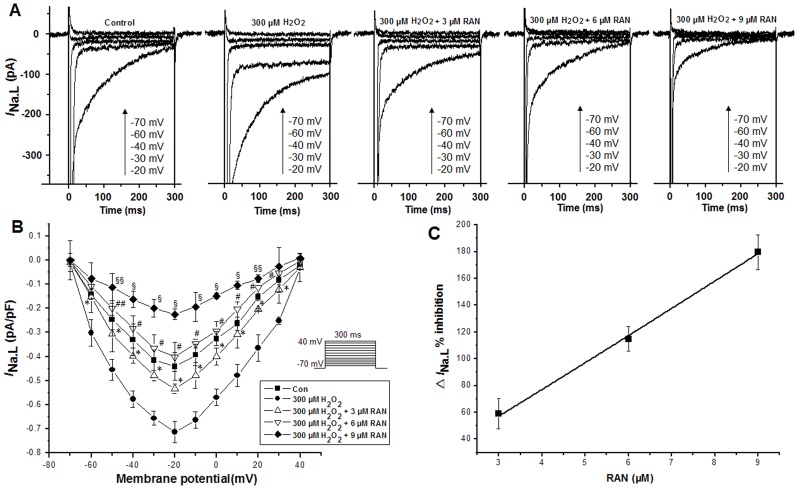
Ranolazine (RAN) inhibited the increase in *I*_Na.L_ caused by 300 µM H_2_O_2_. *A*. 3, 6 and 9 µM ranolazine decreased the amplitudes of the increased *I*_Na.L_ induced by 300 µM H_2_O_2_ in a concentration dependent manner. *B*. The I-V relationships of *I*_Na.L_ after the sequential application of H_2_O_2_ alone, H_2_O_2_ plus 3, 6 and 9 µM ranolazine. *C.* the inhibition amounts of 3, 6 and 9 µM ranolazine on △*I*_Na.L_ induced by 300 µM H_2_O_2_. Values are expressed as mean ± SD, n = 8 cells/group. ^*^P<0.01 versus H_2_O_2_ group; ^#^P<0.01, ^##^P<0.05 versus 3 µM ranolazine group; ^§^P<0.01, ^§§^P<0.05 versus 6 µM ranolazine group.

Currents were recorded using depolarizing pulses with a duration of 300 ms at a rate of 0.25 Hz from a HP of −120 mV, in 10 mV increments between −70 and −20 mV. Administration of resveratrol at concentrations of 10, 20, 40 and 80 µM resulted in a decrease in the amplitudes of *I*_Na.L_ in a concentration dependent manner in myocytes exposed to H_2_O_2_ (Figure 2). H_2_O_2_ (300 µM) increased the amplitudes of *I*_Na.L_ but 10, 20, 40 and 80 µM resveratrol decreased the amplitudes of *I*_Na.L_ in the continued presence of H_2_O_2_ (Figure 2A). Shown in figure 2B are the I-V relationships of *I*_Na.L_ after the sequential application of 300 µM H_2_O_2_, 10, 20, 40 and 80 µM resveratrol respectively, without a shift of the voltage at which the *I*_Na.L_ amplitude was maximal. Figure 2C shows the inhibition amounts of 10, 20, 40 and 80 µM resveratrol on the △*I*_Na.L_ (H_2_O_2_ induced increase in *I*_Na.L_) induced by 300 µM H_2_O_2_ with an IC_50_ of 26.192 µM. Ranolazine (3, 6 and 9 µM) attenuated the increased *I*_Na.L_ in the presence of 300 µM H_2_O_2_ in a concentration dependent manner (Figure 3). Shown in figure 3B are the I-V relationships of *I*_Na.L_ after the sequential application of 300 µM H_2_O_2_ in the absence and presence of 3, 6 and 9 µM ranolazine respectively, without a shift of the voltage at which the *I*_Na.L_ amplitude was maximal. Figure 3C shows the inhibition amounts of 3, 6 and 9 µM ranolazine on the △*I*_Na.L_ induced by 300 µM H_2_O_2_ with 300 ms voltage steps from a HP of −120 to −20 mV with an IC_50_ of 2.457 µM. TTX (4 µM) reversed the increased *I*_Na.L_ caused by 300 µM H_2_O_2_. The values of *I*_Na.L_ under the control conditions, after application of 300 µM H_2_O_2_ and 4 µM TTX were −0.323±0.087, −0.878±0.071(n = 6, P<0.05 versus control) and −0.258± −0.045 pA pF^−1^ (n = 6, P<0.05 versus H_2_O_2_), respectively.

### Effects of Resveratrol, Ranolazine and TTX on Increased Electrogenic *I*_NCX_ by H_2_O_2_

**Figure 4:**
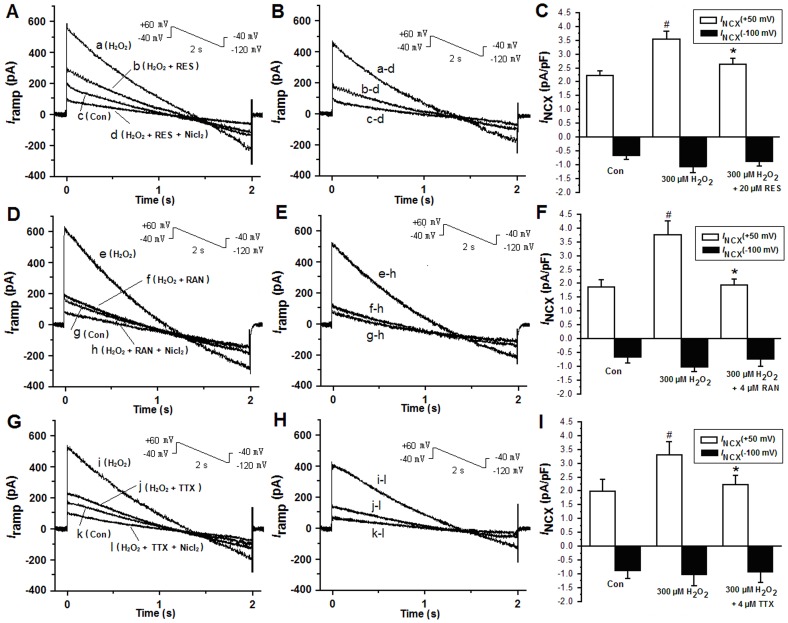
*A*. The I-T (current-time relationship) curves were constructed from the negative slope region of a ramp voltage clamp, and were sequentially shown during no drug (control, trace c), H_2_O_2_ alone (trace a), H_2_O_2_ plus 20 µM resveratrol (trace b), and Ni^2+^ (5 mM) (trace d) in the continued presence of 300 µM H_2_O_2_. *B.* Ni^2+^-sensitive *I*_NCX_ was obtained by subtracting the data in the trace d from the data in trace a, b, and c in panel A. The enhanced reverse *I*_NCX_ induced by H_2_O_2_ was restored by 20 µM resveratrol. *C.* Histograms show the mean current densities of *I*_NCX_ obtained from B. Values are expressed as mean ± SD, n = 8 cells/group. ^#^P<0.01 *versus* control group;^ *^P<0.01 versus H_2_O_2_ group. *D*. Similar to A, the I-T (current-time relationship) curves were constructed, and were shown during no drug (control, trace g), H_2_O_2_ alone (trace e), H_2_O_2_ plus 4 µM RAN (trace f), and Ni^2+^ (5 mM) (trace h) in the continued presence of H_2_O_2_. *E*. Ni^2+^-sensitive *I*_NCX_ were obtained by subtracting the data in trace h from the data in traces e, f and g in panel D. The reverse *I*_NCX_ increased by H_2_O_2_ was restored by 4 µM RAN. *F*. Histograms show the mean current densities of *I*_NCX_ obtained from E. Values are expressed as mean ± SD, n = 8 cells/group. ^#^P<0.01 *versus* control group;^ *^P<0.01 versus H_2_O_2_ group. *G*. Similar to A and D, the I-T (current-time relationship) curves were constructed, and were shown during no drug (control, trace k), H_2_O_2_ alone (trace i), H_2_O_2_ plus 4 µM TTX (trace j), and Ni^2+^ (5 mM) (trace l) in the continued presence of H_2_O_2_. *H*. Ni^2+^-sensitive *I*_NCX_ were obtained by subtracting the data in trace l from the data in traces i, j and k in panel G. The reverse *I*_NCX_ increased by H_2_O_2_ was restored by 4 µM TTX. *I*. Histograms show the mean current densities of *I*_NCX_ obtained from H. Values are expressed as mean ± SD, n = 8 cells/group. ^#^P<0.01 *versus* control group;^ *^P<0.01 versus H_2_O_2_ group.

Electrogenic *I*_NCX_ was measured to determine whether the reverse NCX was activated by the increase of *I*_Na.L_,. Membrane currents were elicited using ramp voltage-clamp pulses from a HP of −40 mV to +60 mV for 100 ms and then ramped to −120 mV over a period of 2 seconds (i.e. at 90 mV s^−1^) before returning to −40 mV. The current-time relationship was constructed from the declining slope of the ramp pulse (Figure 4A, 4B, 4D, 4E, 4G, 4H). Figure 4B, 4E, 4H shows the Ni^2+^-sensitive (NCX) current obtained by subtracting the data in the trace d, h or l from the data in the trace a, e, i, b, f, j, c, g or k in the panel 4A, 4D or 4G. Figure 4C, 4F, 4I are *I*_NCX_ measured at voltage levels of +50 mV and −100 mV, respectively, as the Ni^2+^-sensitive current by subtracting the current recorded in the presence from that in the absence of 5 mM NiCl_2_.

*I*_NCX_ was recorded after 7 minutes of exposure of H_2_O_2_. The mean current density of the inward *I*_NCX_ had little change, while the reverse *I*_NCX_ increased *significantly (*n = 7, Figure 4B, 4C, 4E, 4F, 4H, 4I). 20 µM resveratrol, 4 µM ranolazine or 4 µM TTX diminished the increase of *I*_NCX_ (n = 7, Figure 4B, 4C, 4E, 4F, 4H, 4I).

### Effects of Resveratrol and TTX on Increased Intracellular Ca^2+^ Transient by H_2_O_2_

**Figure 5:**
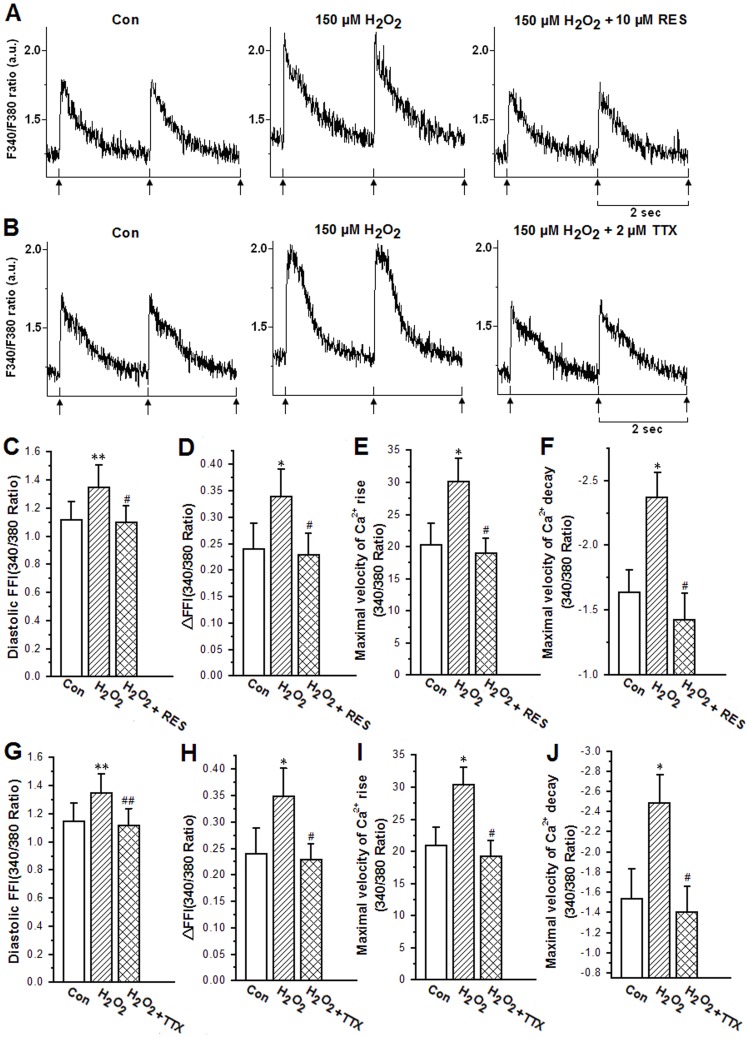
Effects of 10 µM resveratrol and 2 µM TTX on intracellular Ca^2+^ transient properties of adult rabbit ventricular myocytes in the presence of H_2_O_2_ (150 µM). *A, B.* Representative recordings showing intracellular Ca^2+^ transients under different conditions; *C, G.* diastolic intracellular Ca^2+^ fura-2 fluorescence intensity (FFI); *D, H.* electrically stimulated increase in FFI (△FFI); *E, I.* maximal velocity of intracellular Ca^2+^ transient rise; *F, J.* maximal velocity of intracellular Ca^2+^ transient decay. Values are expressed as mean ± SD, n = 6–7 cells/group, **P<0.05, *P<0.01 *versus* control group; ^##^P<0.05;^ #^P<0.01 *versus* H_2_O_2_ group.

As shown earlier, resveratrol could reduce the increase in *I*_Na.L_ and *I*_NCX_ by H_2_O_2_, theoretically it should also decrease the increase in intracellular Ca^2+^ transients by H_2_O_2_. To minimize the cell contracture by 300 µM H_2_O_2_ due to the increase in the amplitude of calcium transients and diastolic calcium concentration, the concentration of H_2_O_2_ used was 150 µM. Cells were perfused with KHB solution for 5 min and then with KHB containing 150 µM H_2_O_2_ for 10 min. The diastolic intracellular Ca^2+^ fura-2 fluorescence intensity (FFI), fura-fluorescence intensity change (△FFI), maximal velocity of Ca^2+^ rise and Ca^2+^ decay were all enhanced by 150 µM H_2_O_2_ (Figure 5). However, 10 µM resveratrol reversed all these enhancements, as shown in Figure 5A, 5C, 5D, 5E, 5F. TTX (2 µM) also depressed these enhancements of the abovementioned parameters induced by 150 µM H_2_O_2_ (Figure 5B, 5G, 5H, 5I, 5J). These results indicated that both resveratrol (10 µM) and TTX (2 µM) could attenuate the H_2_O_2_-induced augmentations in diastolic Ca^2+^ concentration and calcium transients amplitude.

### Effects of TTX on *I*_Ca.L_

**Figure 6:**
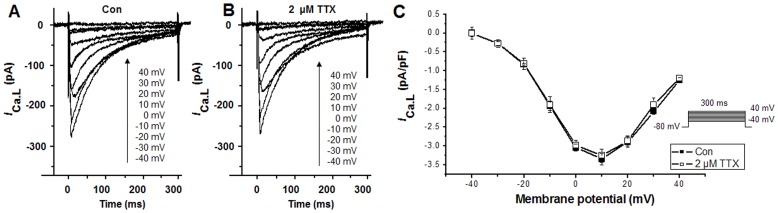
Effects of 2 µM TTX on *I*_Ca.L_ under control conditions in rabbit ventricular myocytes. *A. I*_Ca.L_ under control conditions. *B*. *I*_Ca.L_ after the application of 2 µM TTX. *C*. Effects of TTX (2 µM) on current-voltage relationship of *I*_Ca.L_. Values are expressed as mean ± SD, n = 6 cells/group. P>0.05 versus control group.

Recent evidence suggests a potential for TTX to inhibit L-type Ca^2+^ channels [28]. The results in this study showed that 2 µM TTX inhibited H_2_O_2_-induced augmentations in diastolic Ca^2+^ concentration and amplitude of calcium transients. To identify the effect of 2 µM TTX on intracellular Ca^2+^ was from its blocking of *I*_Na.L_ but not the inhibition of L-type Ca^2+^ channels, *I*_Ca.L_ was measured. The results indicated that at a low concentration (2 µM) TTX is relatively a selective *I*_Na.L_ blocker. Using depolarizing pulses with a duration of 300 ms applied at 0.5 Hz from a HP of −80 mV, in 10 mV increments between −40 and +40 mV, *I*_Ca.L_ was recorded in the absence (Figure 6A) and presence (Figure 6B) of 2 µM TTX. Figure 6C showed the effect of TTX (2 µM) application on the current-voltage relationship of *I*_Ca.L_. TTX at concentration of 2 µM had no effect on *I*_Ca.L_, accounting for that the effects of 2 µM TTX to inhibit H_2_O_2_-induced augmentations in diastolic Ca^2+^ concentration and amplitude of calcium transients were from its inhibition on *I*_Na.L_ and subsequently the reverse *I*_NCX_.

## Discussion

The mechanisms underlying the genesis of ischemia- and reperfusion-induced arrhythmias are notoriously complex and controversial. There has been an interest in the concept that oxygen free radicals play a role in the pathogenesis of myocardial ischemia and infarction. It has been reported that a burst of H_2_O_2_, an important reactive oxygen species, is generated in the myocardium during ischemia and reperfusion [29]–[33] and causes Ca^2+^ overload through many ways [34], [35], [36], [37]. For example, the activation of ryanodine receptors with H_2_O_2_ could also account for the increased cytosolic Ca^2+^ levels found with ROS production, which could account for the Ca^2+^ overload in cells [34]. Furthermore, the excessive amount of H_2_O_2_ could increase *I*_Na.L_ in cardiomyocytes, subsequently leading to intracellular Ca^2+^ overload through reverse NCX (the Na^+^-dependent Ca^2+^ overload induced by *I*_Na.L_) and ultimately causing cell damage [10], [23], [38], [39]. Reducing agent, e.g., dithiothreitol (DTT) and reduced glutathione (GSH), could reverse the increased *I*_Na.L_ by H_2_O_2_ and hypoxia [5], [24], [25]. Resveratrol, a natural antioxidant, has beneficial effects against coronary heart disease. Previous studies have shown that resveratrol effectively suppressed ischemia/reperfusion-induced arrhythmia [40], [41] and reduced both peak *I*_Na_ and *I*_Na.L_ in the R1623Q LQT3 mutation in a recombinant expression system [27]. But the effect of resveratrol on the increased *I*_Na.L_ and reverse *I*_NCX_ under proarrhythmic conditions (H_2_O_2_) in rabbit ventricular myocytes has not been investigated yet. The data from this study addressed the impact of resveratrol on the Na^+^-dependent Ca^2+^ overload.

In this study, *I*_Na.L_ was increased by H_2_O_2_ (Figure 2, 3). Ranolazine attenuated the increased *I*_Na.L_ by H_2_O_2_ in a concentration dependent manner (Figure 3) and 4 µM TTX attenuated the increased *I*_Na.L_ increased by H_2_O_2_ as well. These data are consistent with other reports and our previous studies that the *I*_Na.L_ inhibitors ranolazine and TTX significantly inhibited late *I*_Na.L_ at clinical relevant concentrations [42], [43]. H_2_O_2_-induced intracellular Na^+^ and Ca^2+^ overload was associated with an enhanced *I*_Na.L_ and therefore was attenuated by the *I*_Na.L_ inhibitors ranolazine and TTX [10]. The *I*_Na.L_ blocking agents may be effective in preventing arrhythmias by reducing [Na^+^]_i_ load and subsequently the [Ca^2+^]_i_ load. However, ranolazine has been suggested to inhibit the cardiac ryanodine receptor (IC_50_ = 10 µM) [44], which could also modulate intracellular Ca^2+^ levels. Ranolazine is currently approved as an antianginal agent that reduces the Na^+^-dependent Ca^2+^ overload via inhibition of the *I*_Na.L_ and thus improves diastolic tone and oxygen handling during myocardial ischemia [7]. *I*_Na.L_ is an important contributing factor to intracellular Ca^2+^ overload in the pathogenesis of myocardial ischemia and infarction. In rabbit ventricular myocytes, low concentrations of TTX (1.5–4.0 µM) did not alter the L-type Ca^2+^ current (Figure 6) and *I*_Na.T_ [6], [25], [45], but obviously inhibited the *I*_Na.L_. Accordingly TTX was used to confirm the process of Na^+^-dependent Ca^2+^ overload induced by H_2_O_2_. The effect of resveratrol on *I*_Na.L_ is similar to ranolazine and TTX. Resveratrol inhibited *I*_Na.L_ in both normal and H_2_O_2_-treated cells in a concentration dependent manner (Figure 1, 2). This result is consistent with our previous studies that DTT and reduced glutathione could reverse the increase in *I*_Na.L_ induced by either H_2_O_2_ or hypoxia [24], [25], indicating resveratrol may act as an antioxidant to eliminate the detrimental effects of H_2_O_2_ on *I*_Na.L_. Changes in redox potential or surface charge may account for some ionic current block [46], therefore it is possible that the antioxidant properties of resveratrol may contribute to the *I*_Na.L_ inhibition observed in this study.

Recently, it has been reported that reverse *I*_NCX_ was increased along with the increased *I*_Na.L_ during hypoxia and was decreased along with the *I*_Na.L_ inhibition by TTX in hypoxic ventricular myocytes, suggesting that the increased *I*_Na.L_ contributed to the increase in the reverse *I*_NCX_ [6]. In this study, 300 µM H_2_O_2_ increased the reverse *I*_NCX_ while the inward *I*_NCX_ was not affected obviously, whereas ranolazine or TTX attenuated the increase in the reverse *I*_NCX_ *significantly* (Figure 4). Different from *I*_Na.T_, *I*_Na.L_ can be blocked by a low concentration of ranolazine and TTX, and the consequent reduction of Na^+^ loading via the decrease of the *I*_Na.L_ can prevent the increase in the reverse *I*_NCX_-induced intracellular Ca^2+^ accumulation [47]. Ranolazine (4 µM) and TTX (4 µM) decreased the reverse *I*_NCX_ through the inhibition of *I*_Na.L._ Similarly, resveratrol (20 µM) attenuated the increase in the reverse *I*_NCX_ by H_2_O_2_. Thus, we concluded that the effect of resveratrol to inhibit the increased reverse *I*_NCX_ caused by H_2_O_2_ was from its inhibition of *I*_Na.L_.

In this study, 150 µM H_2_O_2_ significantly increased the amplitude of calcium transients and diastolic calcium concentration in the ventricular cell which could be reversed by TTX (2 µM). The intracellular Ca^2+^ overload caused by ROS was due to an increase in [Na^+^]_i_ followed with an increase in Ca^2+^ influx via the reverse mode of the NCX [48]. Then the large entry of Ca^2+^ into the cell will cause intracellular Ca^2+^ overload [49], [50]. TTX also inhibited L-type Ca^2+^ channel with an IC_50_ value of 55±2 µM [28]. In this study in rabbit ventricular myocytes, 2 µM TTX inhibited *I*_Na.L_ and restrained Ca^2+^ overload induced by H_2_O_2_ but not affected L-type Ca^2+^ channels (Figure 6), supporting that *I*_Na.L_ played an important role in the genesis of Ca^2+^ overload induced by H_2_O_2_. TTX also reversed the increase in calcium transients amplitude and diastolic calcium concentration through inhibiting the increased *I*_Na.L_ by H_2_O_2_. Resveratrol (10 µM) also restrained the increased calcium transients amplitude and the diastolic calcium concentration induced by H_2_O_2_ (150 µM). Therefore the effects of resveratrol on the Na^+^-dependent Ca^2+^ overload induced by enhanced *I*_Na.L_ were similar to 2 µM TTX, suggesting that the reduction of Ca^2+^ overload by resveratrol may have similar mechanism to TTX, i.e., inhibition of *I*_Na.L_. Indeed, resveratrol has also been suggested to inhibit the ryanodine receptor-induced intracellular Ca^2+^ increase [51] which may account for the reduction of [Ca^2+^]_i_. The results in this study indicated that resveratrol reduced both *I*_Na.L_ and reverse *I*_NCX_ which was responsible for the reversal of intracellular Ca^2+^ overload in the presence of H_2_O_2_. Resveratrol may inhibit both the ryanodine receptor-induced intracellular Ca^2+^ overload and *I*_Na.L_-induced increase in reverse *I*_NCX_ to attenuate the intracellular Ca^2+^ overload. Further research will be needed to clarify the contribution of the two pathways by resveratrol in the absence and presence of H_2_O_2_.

### Conclusions

*I*_Na.L_ is an important target for resveratrol to prevent or treat ventricular arrhythmias. *I*_Na.L_ increased by H_2_O_2_ induces intracellular Ca^2+^ overload (the increased diastolic calcium concentration) through the increase in the reverse *I*_NCX_. The inhibitive effect of resveratrol on H_2_O_2_-induced *I*_Na.L_ may reduce the concentration of [Na^+^]_i_, lower [Ca^2+^]_i_ by attenuating reverse NCX to eliminate Ca^+^ overload, and ultimately inhibit the electrical abnormalities.
